# Optimizing dynamic economic dispatch through an enhanced Cheetah-inspired algorithm for integrated renewable energy and demand-side management

**DOI:** 10.1038/s41598-024-53688-8

**Published:** 2024-02-07

**Authors:** Karthik Nagarajan, Arul Rajagopalan, Mohit Bajaj, R. Sitharthan, Shir Ahmad Dost Mohammadi, Vojtech Blazek

**Affiliations:** 1 Department of Electrical and Electronics Engineering, Hindustan Institute of Technology and Science, Chennai, Tamil Nadu India; 2 Centre for Smart Grid Technologies, School of Electrical Engineering, Vellore Institute of Technology, Chennai, Tamil Nadu 600 127 India; 3 Electrical Engineering Department, Graphic Era (Deemed to be University), Dehradun, 248002 India; 4 Hourani Center for Applied Scientific Research, Al-Ahliyya Amman University, Amman, Jordan; 5 Graphic Era Hill University, Dehradun, 248002 India; 6 Applied Science Research Center, Applied Science Private University, Amman, 11937 Jordan; 7 Department of Electrical and Electronics, Faculty of Engineering, Alberoni University, Kapisa, Afghanistan; 8 ENET Centre, VSB—Technical University of Ostrava, 708 00 Ostrava, Czech Republic

**Keywords:** Energy science and technology, Engineering, Mathematics and computing

## Abstract

This study presents the Enhanced Cheetah Optimizer Algorithm (ECOA) designed to tackle the intricate real-world challenges of dynamic economic dispatch (DED). These complexities encompass demand-side management (DSM), integration of non-conventional energy sources, and the utilization of pumped-storage hydroelectric units. Acknowledging the variability of solar and wind energy sources and the existence of a pumped-storage hydroelectric system, this study integrates a solar-wind-thermal energy system. The DSM program not only enhances power grid security but also lowers operational costs. The research addresses the DED problem with and without DSM implementation to analyze its impact. Demonstrating effectiveness on two test systems, the suggested method's efficacy is showcased. The recommended method's simulation results have been compared to those obtained using Cheetah Optimizer Algorithm (COA) and Grey Wolf Optimizer. The optimization results indicate that, for both the 10-unit and 20-unit systems, the proposed ECOA algorithm achieves savings of 0.24% and 0.43%, respectively, in operation costs when Dynamic Economic Dispatch is conducted with Demand-Side Management (DSM). This underscores the advantageous capability of DSM in minimizing costs and enhancing the economic efficiency of the power systems. Our ECOA has greater adaptability and reliability, making it a promising solution for addressing multi-objective energy management difficulties within microgrids, particularly when demand response mechanisms are incorporated. Furthermore, the suggested ECOA has the ability to elucidate the multi-objective dynamic optimal power flow problem in IEEE standard test systems, particularly when electric vehicles and renewable energy sources are integrated.

## Introduction

Fossil fuel-fired power plants continue to be the primary method of generating electric power. The need to investigate alternative energy sources has increased due to the rapid rise in global electricity usage, the continuous depletion of fossil fuel reserves, and the growing environmental impact caused by the burning of fossil fuels in power plants^1,2^. Society's attention has been directed towards sustainable energy solutions due to the urgent need to reduce the negative effects of electricity generation on climate change^3^. Solar and wind power have become noticeable alternatives in this situation, acknowledged for their economic feasibility and ability to meet energy needs without causing harmful emissions^4,5^. However, the incorporation of these environmentally aware energy sources, such as wind and solar technologies, has brought about a level of intricacy and uncertainty in the energy sector. The emerging transition to renewable energy requires a detailed comprehension of the challenges associated to the inherent irregularity and fluctuation of solar and wind resources^6^. This requires a thorough examination of the dynamic properties that arise from integrating these renewable sources into the power grid. As the discussion about sustainable energy progresses, it is crucial to understand the complexities of utilizing solar and wind power to achieve their best possible integration into the overall energy system^7^. These insights are essential for progressing the discussion on sustainable energy usage and developing effective strategies to align the shift towards green energy with the needs of a reliable and robust power grid^8^. The load variation is unaffected by the unpredictability of solar irradiation and wind speed. These resources' unpredictability and sporadic nature present serious obstacles to solving the generation scheduling issue. The inherent variability and irregular characteristics of renewable energy sources, such as wind and solar power, present a potential risk to the stability and dependability of the power grid. This oscillating behavior, commonly known as "blinking," can have detrimental effects on the grid as a whole^9,10^. In order to address these challenges and improve the ability of the power grid to withstand disruptions, the incorporation of pumped hydroelectric energy storage is seen as a feasible solution^11^. Pumped-storage hydropower (PSH) units are widely recognized globally for their ability to effectively manage fluctuations in generation and supply. The growing popularity of PSH units arises from their inherent capacity to efficiently store electrical energy. Pumped-storage hydroelectric (PSH) units play a crucial role in the electric power systems by storing excess electrical energy, which is usually available and cost-effective during low-demand periods, as hydraulic potential energy^12^. This complex procedure entails the movement of water from the lower reservoir of the PSH unit to its upper reservoir. During times of high demand, the stored hydraulic potential energy is used to meet the increased load requirements, thereby assisting to maintain stability in the power grid. PSH units, operating on a daily or weekly basis, provide an efficient solution to mitigate the effects of renewable energy intermittency on the power grid^13^. Implementing Pumped Storage Hydro (PSH) units results in a gradual decrease in the overall fuel expenditure in a power system. The cost-effectiveness of this approach is due to the strategic placement of PSH units, which helps to stabilize fluctuations in energy supply and demand and optimize the operation of the power system^14^. Overall, the integration of pumped hydroelectric energy storage, demonstrated by PSH units, is an effective approach to mitigate the intermittent nature of renewable energy sources. By utilizing the storage capabilities of PSH, the power grid can attain heightened stability, decreased operational expenses, and enhanced flexibility to accommodate the ever-changing landscape of renewable energy generation^15,16^.

A modest sovereign system's optimal generation scheduling using renewable energy sources has been covered in^17^. Although clean and pollution-free, renewable energy sources have a limited ability to provide electricity. The optimal approach to address the economic dispatch quandary lies in dynamic economic dispatch (DED). This approach efficiently distributes the time-varying load demand across all active generating units, while taking into account the limitations presented by thermal generator ramp rates^18^. In the realm of Dynamic Economic Dispatch (DED), decisions made at one time significantly influence subsequent decisions. Addressing this, a novel Enhanced Non-Dominated Sorting Crisscross Optimization (ENSCSO) algorithm was introduced to solve the multi-objective Dynamic Economic Emission Dispatch problem^19^. This algorithm was tested via simulations on a ten-unit generation system that integrates wind power and a time-of-use demand response program. Ameliorated dragonfly algorithm (ADFA) was applied to solve static economic load dispatch and dynamic economic load dispatch problem in^20^. Static economic dispatch was carried out on three different test systems and dynamic economic dispatch was implemented on two different test systems. In^21^, a Levy Interior Search Algorithm was crafted with a focus on resolving the multi-objective economic load dispatch issue, integrating the incorporation of wind power. The objective functions considered were operation cost and system risk. A simulation was conducted using a modified IEEE 30-bus system, incorporating the integration of wind power. A distributed structure and stochastic linear programming game were presented, allowing for the scheduling of appliances and storage units as well for the energy payments in^22^. A distributed primal–dual continuous time consensus algorithm was implemented for solving dynamic economic dispatch problem^23^. Simulation was carried out on three different test systems. An improved version of Circle Search Algorithm was introduced in ref.^24^ to resolve the economic emission dispatch problem by incorporating demand response integration. Improved circle search algorithm was investigated on IEEE 6-bus and IEEE 30-bus system to implemented the multi-objective economic emission dispatch^25^. In^26^, multi-objective particle swarm optimization was proposed to solve the dynamic economic emission dispatch problem. Within the Demand-Side Management (DSM) process, a strategy utilizing day-ahead load shifting techniques was implemented to manage residential loads. The primary objective involved minimizing the utility's energy bill. The application of the Interior Search Algorithm was utilized to address the economic load dispatch problem within a microgrid setting, as referenced in^27^. Multi-objective dynamic optimal power flow problem was implemented using harmony search algorithm. In^27^, the day-ahead load shifting DSM technique was enacted using a day-ahead pricing strategy combined with an energy consumption game. Additionally, in^28^, the successful implementation of the normal boundary intersection method effectively addressed the centralized multi-objective dynamic economic dispatch incorporating demand side management for individual residential loads and electric vehicles. Generation costs, emissions, and energy loss are considered as objective functions. A suite of innovative optimization algorithms was developed to tackle various complex challenges within energy management systems. In^29^, the Improved Mayfly Optimization Algorithm was devised to solve the combined economic emission dispatch problem within a microgrid setting. Ref.^30^ introduced the Chaotic Fast Convergence Evolutionary Programming (CFCEP) aimed at resolving the combined heat and power dynamic economic dispatch problem. This solution incorporated demand side management, renewable energy sources, and pumped hydro energy storage. The Social Group Entropy Optimization (SGEO) technique, highlighted in reference^31^, was proposed to address the fuel-constrained dynamic economic dispatch problem. This strategy combined demand-side management, renewable energy sources, and a pumped hydro storage plant. It implemented a Multi-Objective Dynamic Economic Emission Dispatch by incorporating game theory-based demand response techniques^32^. Lastly, in ref.^33^, a Multi-Objective Dynamic Economic Emission Dispatch approach was applied within a microgrid context. This implementation incorporated demand response strategies along with a zero-balance approach.

As outlined in the International Energy Agency's strategic plan, DSM stands as the major choice for energy policy decisions. DSM programs offer various advantages, such as cost reduction and heightened security within power systems. Here's an overview of the ongoing research contributions in this domain:

1. In our research paper, we introduce the Enhanced Cheetah Optimizer Algorithm (ECOA) to address dynamic economic dispatch while integrating renewable energy sources and demand side management. We've integrated chaotic sine map and levy flight mechanism and into this algorithm to improve solution quality and convergence speed. This learning method involves simultaneously considering an estimate and its opposite counterpart, aiming to refine the current candidate solution more effectively.
2. The inherent variability of wind and solar power generators is depicted through the utilization of the most reliable probability density functions (PDFs).
3. The alteration in the generation costs of wind and solar power in relation to the respective scheduled power adjustments is thoroughly investigated.
4. We subjected our proposed algorithm to a comprehensive evaluation to assess its effectiveness in addressing dynamic economic dispatch challenges associated with pumped-storage hydroelectric units and demand-side management. The ECOA algorithm we introduced plays a vital role in determining optimal times for both pumping water to the upper reservoir and releasing it for power generation, taking into consideration factors such as electricity prices, demand patterns, and the availability of renewable energy.
5. The algorithm we proposed was thoroughly examined for its efficacy in resolving dynamic economic dispatch problems involving unconventional energy sources and demand side management. We compared the optimization results of our proposed algorithm with those obtained using COA and GWO for comprehensive analysis.

## Mathematical formulation of dynamic economic dispatch

The primary aim of integrating renewable energy sources into the Dynamic Economic Dispatch (DED) system is to achieve a dual objective of minimizing two factors simultaneously^34^. The primary objective is to minimize the overall expenses linked to thermal power plants by enhancing their operating efficiency. Furthermore, the integration aims to reduce costs associated with the functioning of wind-power producing units and solar Photovoltaic (PV) facilities. This extensive framework of DED expands its scope to incorporate the integration of pumped hydroelectric energy storage, acknowledging its crucial role in mitigating the intermittent nature of renewable energy sources^35,36^. The study attempts to achieve an efficient and cost-effective balance between traditional and renewable energy sources within the dynamic economic dispatch framework using this integrated method^37^.

The formulation of the DED problem with DSM encompasses defining the resultant objective function along with its associated constraints. The fuel cost function for the *i*th thermal generator at time $t$, accounting for the valve-point effect^38,39^, is expressed as:

1 $$F\left( {P_{G} } \right) = \mathop \sum \limits_{i = 1}^{{N_{TH} }} (a_{i} + b_{i} P_{Gi} + c_{i} P_{Gi}^{2} ) + |e_{i} {\text{sin}}(f_{i} *\left( {P_{Gimin} - P_{i} } \right)|$$

where $a_{i}$, $b_{i}$ and $c_{i}$ are fuel cost coefficients of $i^{th}$ generator, k is the total number of generating units, $P_{Gi}$ is the output power of the $i^{th}$ generator in megawatts. Here $e_{i}$ and $f_{i}$ represents the generating cost coefficients of the $i^{th}$ unit are utilized to model valve point loading effect.

### Modelling the costs of renewable energy sources

#### Assessment of direct costs for wind and solar photovoltaic power

The functioning of energy generation from RESs doesn't require any fuel. Hence, in cases where Independent System Operators (ISO) own Renewable Energy Sources (RESs), only maintenance costs are incurred without any associated cost function^40^. Yet, if private organizations manage RESs, the ISO compensates them as per the mutually agreed-upon contract for the scheduled electricity generation^40^.

The assessment of direct costs for wind turbines and solar photovoltaic (PV) power involves a detailed examination of the expenses associated with the design, construction, installation, operation, and maintenance of these renewable energy systems^41,42^. Direct costs are those directly attributable to the development and operation of the specific technology.

The literature offers the direct cost function for the $ith$ wind farm concerning the planned power^40^.

2 $$C_{W} \left( {P_{W} } \right) = K_{W} P_{W}$$

Here, $P_{W}$ represents the generated power and $K_{W}$ represents the direct cost coefficient related to the wind turbine. In connection with is, the direct cost involved in solar PV with scheduled power $P_{PV}$ and cost coefficient, $K_{PV}$ is represented by the following equation

3 $$C_{PV} \left( {P_{PV} } \right) = K_{PV} P_{PV}$$

In this context, $P_{PV}$ denotes the generated power, while $K_{PV}$ represents the direct cost coefficient associated with solar photovoltaic generation.

#### Assessment of reserve cost and penalty cost associated with wind power

As wind energy is inherently unpredictable, the power generated by wind turbines fluctuates over time, potentially surpassing or falling short of the scheduled power^43^. Therefore, the ISO needs to have backup generating capacity to meet demand. The assessment of reserve cost and penalty cost associated with wind power involves evaluating the expenses and penalties incurred due to the intermittent and variable nature of wind energy. Reserve costs and penalty costs are critical aspects in the economic evaluation and operational planning of power systems that include wind power^44^.

The reserve cost for the wind unit is presented based on the literature^40^.

4 $$\begin{aligned} C_{{R_{W,i} }} \left( {P_{W sh,i} - P_{W ac,i} } \right) & = k_{rw,i}^{\prime } \left( {P_{W sh,i} - P_{W ac,i} } \right) \\ & = k_{rw,i}^{\prime } \mathop \smallint \limits_{0}^{{P_{W sh,i} }} \left( {P_{W sh,i} - p_{w,i} } \right)f_{w} \left( {p_{w,i} } \right)dp_{w,i} \\ \end{aligned}$$

When wind generators produce more output power than is scheduled, the ISOs must pay the fine by reducing the power of thermal generators when they do not consume the extra power.

5 $$\begin{aligned} C_{{P_{W,i} }} \left( {P_{W\; ac,i} - P_{W\; sh,i} } \right) & = k_{rw,i}^{\prime } \left( {P_{W\; ac,i} - P_{W\; sh,i} } \right) \\ & = k_{pw,i}^{\prime } \mathop \smallint \limits_{{P_{W\; sh,i} }}^{{P_{W r,i} }} \left( {p_{w,i} - P_{W\; sh,i} } \right)f_{w} \left( {p_{w,i} } \right)dp_{w,i} \\ \end{aligned}$$

Here $P_{W \;sh,i}$ represents the scheduled wind power and $P_{W\; ac,i}$ represents the actual power generated by the wind turbine. The rated power is represented by $P_{W\; r,i}$ while the Probability Density Function (PDF) of the wind power is represented as $f_{w} \left( {p_{w,i} } \right)$. Accordingly, it is possible to compute the reserve and penalty costs for solar-only and solar-and-hydro combined generators^45^. The required input data for modeling the cost of renewable energy sources is obtained from existing literature^30^.

#### Assessment of the penalty and reserve cost associated with PV power

Assessing the cost of generating wind power aligns closely with formulating the stochastic generation cost of solar PV electricity^40^. Moreover, the lognormal Probability Density Function (PDF) proves useful in representing solar radiation^40^. Additionally, reserve and penalty cost models for solar PV-powered plants are devised based on the methodology outlined in Reference^46^. Section 2.3 does the computation for the solar photovoltaic unit's generated power output. The assessment of reserve cost and penalty cost associated with photovoltaic (PV) power involves evaluating the expenses and penalties incurred due to the intermittent and variable nature of solar energy. Reserve costs and penalty costs are critical aspects in the economic evaluation and operational planning of power systems that include solar PV.

The reserve cost for overestimating solar PV power is characterized as^40^:

6 $$\begin{aligned} C_{{R_{PV,i} }} \left( {P_{PV\; sh,i} - P_{S ac,i} } \right) & = k_{rpv,i}^{\prime } \left( {P_{PV\; sh,i} - P_{PV\; ac,i} } \right) \\ & = k_{rpv,i}^{\prime } f_{pv} \left( {P_{PV\; ac,i} < P_{PV\; sh,i} } \right)\left[ { P_{PV\; sh,i} - E\left( {P_{PV\; ac,i} < P_{PV\; sh,i} } \right) } \right] \\ \end{aligned}$$

where $P_{PV\; ac,i}$ represents the actual power generated by the solar PV plant, and $k_{rpv,i}^{\prime }$ denotes the reserve cost coefficient related to the solar PV plant. The expectation of solar PV power below P_ is represented by $E\left( {P_{PV \;ac,i} < P_{PV\; sh,i} } \right)$, and the likelihood of a solar power shortage from the scheduled solar PV power is given by $f_{pv} \left( {P_{PV\; ac,i} < P_{PV\; sh,i} } \right).$ The cost of the penalty for underestimating solar PV power is characterised as^40^:

7 $$\begin{aligned} C_{{P_{PV,i} }} \left( {P_{PV\; ac,i} - P_{PV\; sh,i} } \right) & = k_{ppv,i}^{\prime } \left( {P_{PV \;ac,i} - P_{PV\; sh,i} } \right) \\ & = k_{ppv,i}^{\prime } f_{pv} \left( {P_{PV\; ac,i} > P_{PV\; sh,i} } \right)\left[ { E\left( {P_{PV\; ac,i} > P_{PV\; sh,i} } \right) - P_{PV \;sh,i} } \right] \\ \end{aligned}$$

where $k_{ppv,i}^{\prime }$ represents the penalty cost coefficient for the solar PV plant and $f_{pv} \left( {P_{PV\; ac,i} > P_{PV \;sh,i} } \right)$ characterizes the likelihood that solar power will be above the scheduled power ($P_{PV \;sh,i}$), and $E\left( {P_{PV\; ac,i} > P_{PV\; sh,i} } \right)$ indicates the expectation that solar PV power will be above $P_{PV \;sh,i}$.

#### Formulation of overall generation cost with the integration of renewable energy sources

The overall operation cost is a critical metric that reflects the economic efficiency of the power system operation. The overall operation cost considers the intermittent nature of renewable energy sources, accounting for periods of high and low generation, and the associated economic implications.

The overall operation cost within the DED problem is structured as follows^30,40^:

Minimize

8 $$\begin{aligned} F_{C} & = F\left( {P_{G} } \right) + \mathop \sum \limits_{i = 1}^{{N_{WG} }} \left[ { C_{W} \left( {P_{W} } \right) + C_{{R_{W,i} }} \left( {P_{W\; sh,i} - P_{W \;ac,i} } \right) + C_{{P_{W,i} }} \left( {P_{W \;ac,i} - P_{W \;sh,i} } \right)} \right] \\ & \quad + \mathop \sum \limits_{i = 1}^{{N_{PV} }} \left[ {\left( {C_{PV} (P_{PV} } \right) + C_{{R_{PV,i} }} \left( {P_{PV\; sh,i} - P_{PV \;ac,i} } \right) + C_{{P_{PV,i} }} \left( {P_{PV\; ac,i} - P_{PV\; sh,i} } \right)} \right] \\ \end{aligned}$$

### Equality and inequality constraints

#### Equality constraints

##### Generator power output constraint

The total power generation, when combined with demand-side management, can be expressed through the following Eq^30^:

9 $$\mathop \sum \limits_{i = 1}^{{N_{TH} }} (P_{Git} ) + \mathop \sum \limits_{i = 1}^{{N_{W} }} (P_{Wit} ) + \mathop \sum \limits_{i = 1}^{{N_{PV} }} (P_{PVit} ) + \mathop \sum \limits_{i = 1}^{{N_{pump} }} (P_{GHit} ) = \left( {1 - DR_{t} } \right) \times L_{Base,t} + L_{{S_{t} }} + P_{loss}$$

10 $$\mathop \sum \limits_{i = 1}^{{N_{TH} }} (P_{Git} ) + \mathop \sum \limits_{i = 1}^{{N_{W} }} (P_{Wit} ) + \mathop \sum \limits_{i = 1}^{{N_{PV} }} (P_{PVit} ) - \mathop \sum \limits_{i = 1}^{{N_{pump} }} (P_{PHit} ) = \left( {1 - DR_{t} } \right) \times L_{Base,t} + L_{{S_{t} }} + P_{loss}$$

where $N_{TH}$, $N_{W}$, $N_{PV}$ and $N_{pump}$ denotes the quantity of thermal power units, wind power units, solar photovoltaic units, and pumped storage units, respectively. The power generated by the ith thermal, wind, solar photovoltaic, and pumped storage units is represented as $P_{Gi}$, $P_{Wi}$, $P_{PVi}$ and $P_{GHi}$ respectively.

In order to achieve optimal economic load dispatch, one must include transmission line losses. The transmission line losses are calculated using Newton–Raphson methods and B-coefficient methods. In order to calculate the active power loss $P_{loss} .$ Newton–Raphson method is used in conjunction with the power flow solution. The subsequent equation defines the actual power loss while adhering to equality prerequisites^40^.

11 $$P_{Gj} - P_{Dj} - V_{j} \mathop \sum \limits_{j = 1}^{NB} V_{k} \left[ {G_{jk} \cos \left( {\delta_{j} - \delta_{k} } \right) + B_{jk} sin\left( {\delta_{j} - \delta_{k} } \right)} \right] = 0$$

12 $$Q_{Gj} - Q_{Dj} - V_{j} \mathop \sum \limits_{j = 1}^{NB} V_{k} \left[ {G_{jk} \sin \left( {\delta_{j} - \delta_{k} } \right) + B_{jk} cos\left( {\delta_{j} - \delta_{k} } \right)} \right] = 0$$

With $j = 1, 2, \ldots NB$; in this case, $NB$ represents the total number of buses. $V_{j}$ and $V_{k}$ represents the $jth$ bus and $kth$ bus voltage respectively. $Q_{gj}$ denotes the $jth$ bus reactive power output and $\delta_{j}$ and $\delta_{k}$ characterizes the voltage angle at bus $j$ and bus $k$ respectively.$B_{jk}$ and $G_{jk}$ represents the transfer susceptance and conductance between buses j and $k$ respectively. $P_{Dj}$ and $Q_{Dj}$ represents the $jth$ bus active and reactive power load respectively. In order to determine the equality constraints, the Newton–Raphson load flow technique solution is used. Bus voltage magnitudes and angles can be determined using the power flow solution.

#### Inequality constraints

##### Limits on the lowest and highest generation capacities

Each generator's active power generation output needs to stay within specific minimum and maximum limits^40^. Power generation constraints refer to the limitations and restrictions imposed on the operation of power generation units over time. These constraints are crucial for ensuring the secure and reliable operation of the power system.

13 $$P_{Gimin} \le P_{Gi} \le P_{Gimax} \forall i \in N_{TH}$$

14 $$P_{w}^{min} \le P_{w} \le P_{w}^{max}$$

15 $$P_{PV}^{min} \le P_{PV} \le P_{PV}^{max}$$

##### Pumped-storage constraints

The integration of pumped-storage hydro units adds a dynamic and flexible component to the system, enabling better balancing of supply and demand.

The net water usage of the pumped-storage hydropower (PSH) unit should balance out to zero as the final and initial water volumes in the upper reservoir are considered equal within this scenario^30^.

16 $$V_{{res,j\left( {t + 1} \right)}} = V_{res,jt} + Q_{phjt} \left( {P_{phjt} } \right), j \in N_{pump} , t \in T_{pump}$$

17 $$V_{{res,j\left( {t + 1} \right)}} = V_{res,jt} - Q_{ghjt} \left( {P_{ghjt} } \right), j \in N_{pump} , t \in T_{gen}$$

18 $$P_{ghj}^{min} \le P_{ghj} \le P_{ghj}^{max} , j \in N_{pump} , t \in T_{gen}$$

19 $$P_{phj}^{min} \le P_{phj} \le P_{phj}^{max} , j \in N_{pump} , t \in T_{pump}$$

20 $$V_{res,j}^{min} \le V_{res,jt} \le V_{res,j}^{max} , j \in N_{pump} , t \in T$$

Given the equality between the initial and final water volumes of the upper reservoir in the pumped-storage hydroelectric (PSH) unit for this scenario, the total net water used by the PSH unit should equate to zero^30^.

21 $$V_{res,j0} = V_{res,jT} = V_{res,j}^{start} = V_{res,j}^{end}$$

22 $$\begin{aligned} Q_{net,spent,j} & = Q_{spent,TOT,j} - Q_{pump,TOT,j} \\ & = \mathop \sum \limits_{{t \in T_{gen} }} Q_{ghjt} \left( {P_{ghjt} } \right) - \mathop \sum \limits_{{t \in T_{pump} }} Q_{phjt} \left( {P_{phjt} } \right) = 0 \\ \end{aligned}$$

##### Ramp rate limits of thermal generator

The ramp rate limits of thermal generators are crucial parameters in power system operation and control. The ramp rate refers to the maximum rate at which the power output of a generator can change over a specified time interval. Rapid and large changes in power output from generators can lead to instability in the power grid. By imposing ramp rate limits, the system operators ensure that the changes in power output are gradual, helping to maintain grid stability.

23 $$P_{Git} - P_{{Gi\left( {t - 1} \right)}} \le UR_{i} , i \in N_{t} , t \in T$$

24 $$P_{{Gi\left( {t - 1} \right)}} - P_{Git} \le DR_{i} , i \in N_{t} , t \in T$$

### Wind, solar and hydro uncertainty models

To represent the unpredictable output power from Renewable Energy Sources (RESs), a range of Probability Density Functions (PDFs) are utilized.

The wind speed determines how much power the wind turbines can produce. According to past research investigations^40,46^, the likelihood of wind speed follows Weibull PDF.

The Weibull distribution is commonly used in the field of wind energy because it is well-suited for modeling the variability of wind speeds at a particular location.

25 $$f_{wv} \left( v \right) = \left( {\frac{\alpha }{\lambda }} \right)\left( {\frac{v}{\lambda }} \right)^{{\left( {\alpha - 1} \right)}} exp[^{{ - \left( {\frac{v}{\lambda }} \right)}} ]^{\alpha } \;for\; 0 < v < \infty$$

where $\alpha$ represents the scale of the Weibull PDF and stands for the shape parameter of the Weibull PDF. These variables' values were collected from^30^. Weibull PDF's median is provided by:

26 $$M_{w} = \lambda *\Gamma \left( {1 + \alpha^{ - 1} } \right)$$

The gamma ($\Gamma )$ function is crucial in the context of the Weibull probability density function (PDF) for wind distribution because it is used to normalize the Weibull distribution and ensure that it integrates to 1 over its entire range.

$\Gamma$ function can be represented as:

27 $$\Gamma \left( {x^{\prime } } \right) = \mathop \smallint \limits_{0}^{\infty } e^{ - t} t^{{x^{\prime } - 1}} dt$$

**Figure 1:**
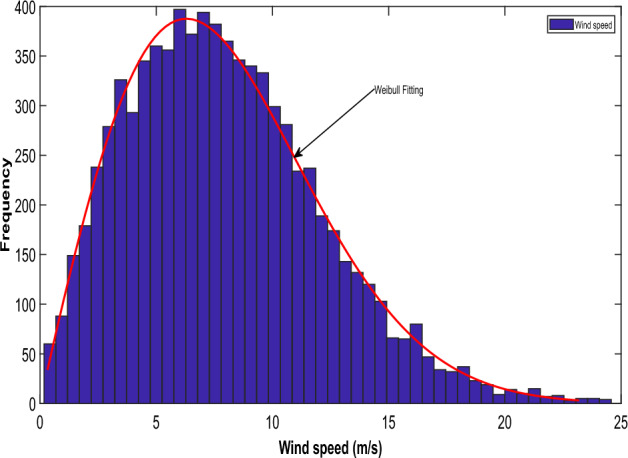
Wind speed variation in wind power generation unit.

As shown in Fig. 1, the frequency distribution is derived from Weibull fitting using wind speed results obtained through simulating 8000 Monte Carlo scenarios. The values for the scale and shape parameters are sourced from^30^. Consistent with the literature^30^, the PDF parameter values have been selected. Achieving a cumulative rated output of 175 MW involves the collective output from 35 wind generators, each possessing a capacity of 5 MW. The subsequent equation delineates the power generated by the wind turbines, contingent upon the wind speed.

28 $$P_{WG} = \left\{ {\begin{array}{*{20}l} 0 \hfill & {for\; v \le v_{in} } \hfill \\ {P_{{W_{r} }} \left( {\frac{{v - v_{in} }}{{v - v_{out} }}} \right)} \hfill & {for \;v_{in} \le v \le v_{r} } \hfill \\ {P_{{W_{r} }} } \hfill & {for \;v_{r} \le v \le v_{out} } \hfill \\ \end{array} } \right.$$

where $P_{{W_{r} }}$ denotes the rated power of a single turbine. $v_{in}$ signifies the cut-in speed, $v_{out}$ denotes the cut-out speed whereas $v_{r}$ is the rated speed. The study investigated different Weibull parameters that dictate the distribution of wind speeds, in line with the selections made in previous studies^30^. Equation (40) emphasizes the discrete nature of the wind generator's output power, notably in specific regions. Specifically, wind farm output remains at zero when wind speed falls below the cut-in speed or exceeds the cut-out speed. The wind generators operate at their rated power within the range delineated between the cut-out and cut-in regions. Previous studies^30,40^ detail the probability associated with these discrete zones.

29 $$f_{{P_{WG} }} = 1 - {\text{exp}}\left[ { - \left( {\frac{{v_{in} }}{\lambda })^{\alpha } } \right] + {\text{exp}}} \right[ - \left( {\frac{{v_{out} }}{\lambda })^{\alpha } } \right]\;for\; \left( {P_{WG} = 0} \right)$$

30 $$f_{{P_{WG} }} = {\text{exp}}\left[ { - \left( {\frac{{v_{r} }}{\lambda })^{\alpha } } \right] - {\text{exp}}} \right[ - \left( {\frac{{v_{out} }}{\lambda })^{\alpha } } \right]\;for\; \left( {P_{WG} = P_{WR } } \right)$$

In the continuous domain, the probability distribution for wind power is expressed as follows^40,46^:

31 $$f_{{P_{WG} }} = \frac{{\alpha \left( {v_{r} - v_{in} } \right)}}{{\lambda^{\alpha } *P_{wr} }}\left[ {v_{in} + \frac{{P_{WG } }}{{P_{wr} }}\left( {v_{r} - v_{in} } \right)} \right]^{\alpha - 1} exp - \left( {\frac{{(v_{in} + \frac{{P_{WG } }}{{P_{wr} }}\left( {v_{r} - v_{in} } \right)}}{\lambda }} \right)^{\alpha }$$

This Weibull PDF is utilized to characterize and model the probability distribution of wind speeds, which is crucial for assessing the potential power output of wind turbines.

Furthermore, the solar photovoltaic (PV) output power is solely contingent on solar irradiance (G), conforming to the parameters of the lognormal Probability Density Function (PDF)^40,46^. A previous study^40^ outlined the probability distribution of solar irradiance, specifying its mean and standard deviation. The lognormal distribution is often used in PV modeling because it provides a good fit for the skewed and positive-valued nature of solar irradiance and power output data. Many natural processes, including solar irradiance, exhibit lognormal characteristics, making the lognormal distribution a suitable choice for modeling. The lognormal distribution is well-suited for data with a positively skewed distribution, capturing the asymmetric behavior often observed in solar irradiance data. The parameters in the lognormal distribution have physical interpretations, such as the mean and standard deviation, which can provide insights into the characteristics of the solar resource.

32 $$f_{PV} \left( G \right) = \frac{1}{{G\sigma \sqrt {2\pi } }}\exp \left[ { - \frac{{\left( {\ln x - \mu } \right)^{2} }}{{2\sigma^{2} }}} \right] \;for\; G > 0$$

The subsequent equation represents the mean of the lognormal distribution ($M_{Lgn}$)

33 $$M_{Lgn} = \exp \left( {\mu + \frac{{\sigma^{2} }}{2}} \right)$$

**Figure 2:**
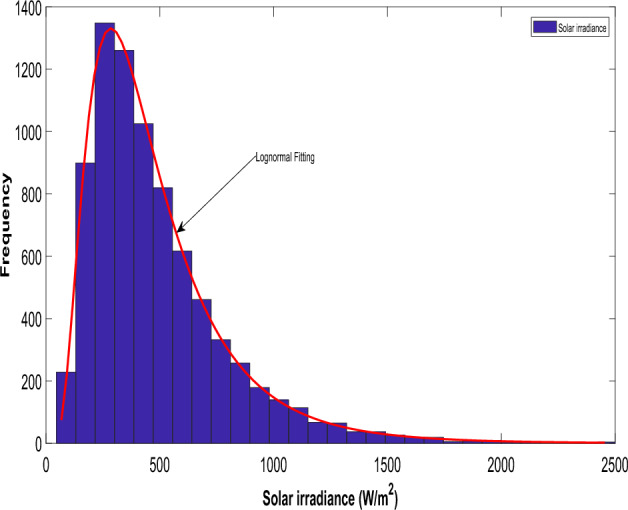
Distribution of solar irradiance for solar PV.

After running 8,000 Monte Carlo simulations, a frequency distribution for solar irradiance is derived, and Fig. 2 illustrates the lognormal fitting, demonstrating the solar PV output power.

34 $$P_{PV} \left( G \right) = \left\{ {\begin{array}{*{20}l} {P_{PVr} \left( {\frac{{G^{2} }}{{G_{std} R_{C} }}} \right)} \hfill & {for\; 0 \le G \le R_{C} } \hfill \\ {P_{PVr} \left( {\frac{G}{{G_{std} }}} \right)} \hfill & { for\; G \ge R_{C} } \hfill \\ \end{array} } \right.$$

The critical value ($$R_{C} )$$ introduces a threshold beyond which the model transitions to a simpler form. This threshold may represent a point where the PV system behavior changes, possibly due to system constraints, saturation effects, or other factors.

In the standard environmental conditions, standard deviation of solar irradiance is represented by $G_{std}$ and certain irradiance is characterized by $R_{C}$. The assumed value for $G_{std}$ stands at 1000 W/m^2^, whereas for $R_{C}$, it amounts to 150 W/m^2^. Regarding the PV module, the rated output power $P_{PVr}$ is specified as 175 MW.

### Demand-side management

DSM initiatives bring forth numerous benefits such as cost efficiency and improved power system security^47^. These programs encompass various categories, prominently featuring demand response. Among these, the time-of-use (TOU) program^48^ stands out—it redistributes a segment of the load demand from peak hours to off-peak periods or times of lower cost, while maintaining the overall load demand. This TOU program served as the foundational inspiration for the demand response program applied in this study. This flattens the load curve and lowers the expected operation cost. The numerical model for the TOU program is created in line with Eq. (35) and is constrained by Eqs. (36) - (39).

35 $$L_{t} = \left( {1 - DR_{t} } \right) \times L_{Base} + L_{st}$$

36 $$\mathop \sum \limits_{t = 1}^{T} L_{st} = \mathop \sum \limits_{t = 1}^{T} DR_{t} \times L_{Base,t}$$

37 $$L_{{Inc_{t} }} = Inc_{t} \times L_{Base,t}$$

38 $$DR_{t} \le DR^{max} , t \in T$$

39 $$Inc_{t} \le Inc^{max} , t \in T$$

## Enhanced Cheetah optimizer algorithm

Akbari et al.^49^ introduced the COA algorithm, drawing inspiration from the hunting techniques of cheetahs. This method integrates three primary strategies: prey search, ambush tactics, and active attacks. Significantly, it implements a mechanism to navigate away from a prey location and return to a home position, effectively avoiding entrapment in local optimal points. Each cheetah's potential hunting patterns correspond to potential solutions for the problem at hand. The algorithm operates on the premise that the population's best position determines the optimal solution, akin to identifying the prey. Cheetahs adapt their hunting patterns to enhance their performance over the hunting period. By mimicking these strategies, the COA algorithm^49^ effectively seeks optimal solutions for intricate problems.

When a cheetah scans its surroundings, it can detect potential prey, giving it the option to either wait for the prey to approach or to initiate an immediate attack upon spotting it. The attack itself involves two distinct phases: a rapid approach followed by capture. However, several factors might prompt the cheetah to abandon the hunt, such as low energy reserves or if the prey is too agile. In such scenarios, the cheetah might retreat to its resting spot, preparing for a fresh hunting opportunity. The cheetah carefully assesses the prey's condition, the environment, and the distance involved before choosing between these strategies. The COA algorithm encapsulates this entire hunting process, relying on the strategic utilization of these tactics across multiple hunting cycles or iterations^49^. Essentially, the COA algorithm leverages these intelligent hunting strategies iteratively throughout the hunting process.

i. Searching: Cheetahs engage in scanning or active search within their territories or the surrounding area to locate prey within the search space.
ii. Sitting-and-waiting: Upon detecting prey but under unfavorable conditions, cheetahs may opt to sit and wait, allowing the prey to approach or for a better opportunity to arise.
iii. Attacking: This strategy involves two crucial phases:
  - Rushing: Once committed to an attack, cheetahs sprint toward the prey at maximum speed.
  - Capturing: Leveraging speed and agility, cheetahs capture the prey by closing in swiftly.
iv. Returning home and leaving prey: This strategy comes into play under two circumstances. Firstly, if the cheetah fails to catch its prey, it may choose to relocate or return to its territory. Secondly, when a certain time lapses without successful hunting, the cheetah may reposition itself to the last known prey location and conduct further searches in that area^49^. Detailed mathematical models for these hunting strategies are expounded upon in subsequent sections.

The CO algorithm has demonstrated strong capabilities in tackling expansive problems. However, as the upcoming experimental results will demonstrate, there remains an opportunity for improvement in terms of convergence speed and computational time, particularly when fine-tuning the parameters of photovoltaic models. To overcome these limitations, we present an upgraded iteration of the COA algorithm tailored explicitly to tackle these drawbacks.

### Searching strategy

In the exploration phase of the COA algorithm, each cheetah adjusts its position by referencing its prior location. Cheetahs commonly follow the lead of the leader within their group. Expanding upon this notion, the search approach detailed in Eq. (16) is adapted based on the position of the group's second-best cheetah, designated as $X_{L,j}^{t}$, influencing the modification process. This adjustment is detailed as follows^50^:

40 $$X_{i,j}^{t + 1} = X_{L,j}^{t} + \hat{r}^{t} \cdot \alpha_{i,j}^{t}$$

where the randomization parameter ($\hat{r}^{t}$) and the random step length ($\alpha_{i,j}^{t}$) undergo modifications as follows:

The value of the randomization parameter ($\hat{r}^{t}$) in Eq. (40) can be ascertained through the implementation of a sine map, where the initial values for $C_{t}$ and $a$ are specifically set at 0.36 and 2.8 as indicated in reference^51^.

41 $$C_{t + 1} = \frac{a}{4}\sin \left( {\pi C_{t} } \right), 0 < a < 4$$

where $t$ represents the current iteration number.

The random step length ($\alpha_{i,j}^{t}$) can be represented as

42 $$\alpha_{i,j}^{t} = X_{{k^{\prime } ,j}}^{t} - X_{{i^{\prime } ,j}}^{t}$$

Here $X_{{k^{\prime},j}}^{t}$ and $X_{{i^{\prime},j}}^{t}$ are the positions of $kth$ and $ith$ cheetahs in the sorted population, respectively.

Emphasizing the alignment of every cheetah's position around the group leader plays a crucial role in the local search phase. Furthermore, the second term in Eq. (40) enhances solution diversity, actively aiding in the global search or exploitation phase. In addition, introducing substantial strides during the hunting phase via the random parameter extends solutions beyond variable ranges. Subsequently, these are substituted by fresh random solutions within the population. This dual purpose not only broadens the spectrum of solutions but also shields the algorithm from being stuck in local optimum points.

### Attacking strategy

To bolster the optimization capabilities of COA algorithm, the researcher crafted the Enhanced Cheetah Optimizer (ECOA) algorithm. This new approach combines principles inspired by Levy flights, mirroring the flight patterns observed in birds. Adopting a Levy flight-based approach for system identification offers expedited convergence without relying on derivative information^40^. This method employs stochastic random searches based on Levy flight concepts^52^. Integrating the Levy flight approach bolsters local search capabilities, mitigating the risk of local entrapment for the optimal solution^52^.

Furthermore, the attacking strategy within the ECOA algorithm undergoes reformulation as follows^40^:

43 $$X_{i,j}^{t + 1} = X_{B,j}^{t} + Levy\left( \lambda \right) \cdot \beta_{i,j}^{t}$$

44 $$Levy\left( \lambda \right) = 0.01 \frac{{r_{1} \sigma }}{{\left| {r_{2} } \right|^{{\frac{1}{\beta }}} }}$$

where σ can be calculated as^40^:

45 $$\sigma = [\Gamma \left( {1 + \lambda } \right){\text{sin}}\left( {\pi \frac{\lambda }{2}} \right)/\left( {\Gamma \left( {\frac{1 + \lambda }{2}} \right)\lambda \left[ {2^{{\frac{{\left( {\lambda - 1} \right)}}{2}}} } \right]} \right)]^{1/\lambda }$$

The function $\Gamma \left( x \right)$ represents the factorial of (x-1), while $r_{1}$ and $r_{2}$ denote indiscriminate numbers within the range of $\left[ {0,1} \right].$ For $1 < \beta \le 2$, a constant value (e.g., 1.5) for $\beta$ is specifically applied in this research^49^. The symbol $Levy\left( \lambda \right)$ signifies step length, employing the Levy distribution characterized by infinite variance and a mean of $1 < \lambda < 3$. $\lambda$ serves as the distribution factor, with $\Gamma \left( . \right)$ representing the gamma distribution function.

Within the COA algorithm, the interaction factor considers the position of neighbouring cheetahs. Ordinarily, cheetahs hunt individually, adapting their positions in response to their prey's whereabouts. Therefore, in this newly suggested attack strategy, each cheetah adjusts its position relative to the prey during the attack phase, advancing toward it following this formula^50^:

46 $$\beta_{i,j}^{t} = X_{B,j}^{t} - X_{i,j}^{t}$$

**Figure 3:**
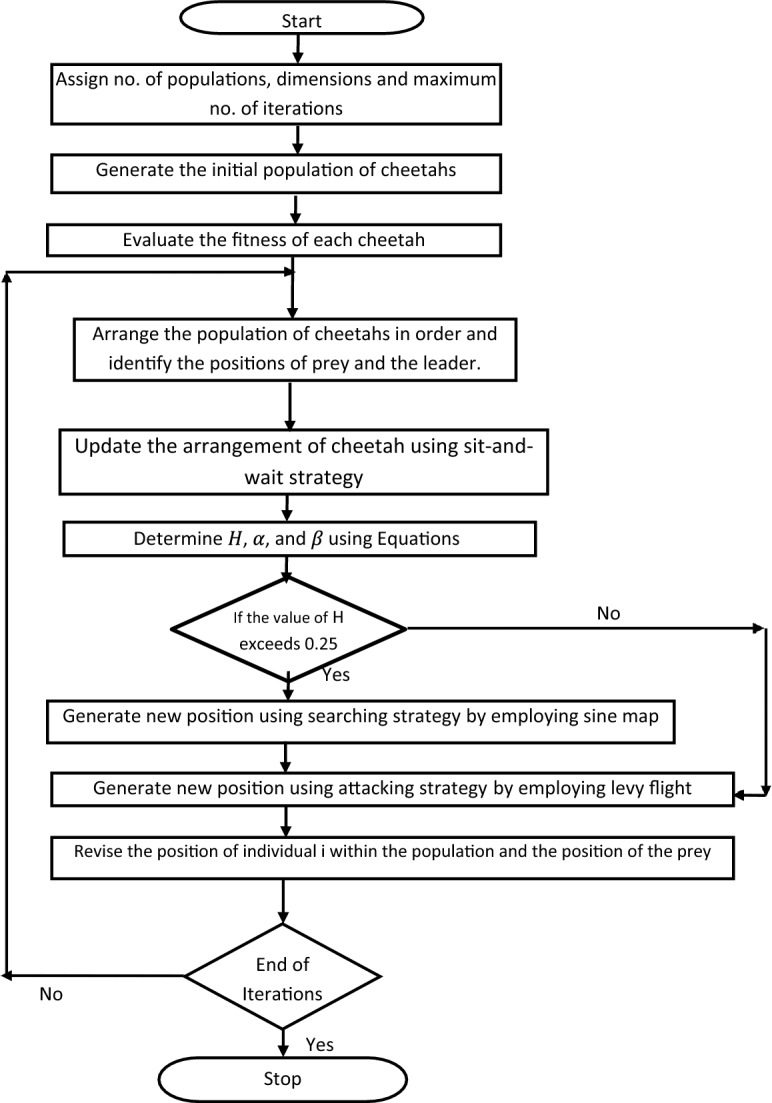
Flowchart of the proposed enhanced cheetah optimizer algorithm.

This refined attack strategy significantly accelerates the COA algorithm's ability to approach near-optimal solutions swiftly. It bolsters the algorithm's local search prowess (exploitation phase), thus amplifying its convergence speed. Figure 3 showcases the schematic of the enhanced Cheetah Optimizer Algorithm as proposed.

## Results and discussion

### Test system: I

The proposed approach has been deployed to address the dynamic economic dispatch problem, both with and without DSM. To gauge its effectiveness, optimization outcomes were compared against COA, GWO, CFCEP^30^, FCEP^30^, CCDE^30^, and HSPSO^30^. The MATLAB 9.12 software was utilized to implement the ECOA, COA, and GWO^53^ models on a Laptop with an AMD Athlon processor, 1 TB storage, and 3.0 GHz processing speed. The test system encompasses 10 thermal power plants, one equivalent wind turbine, a solar photovoltaic plant, and a pumped-storage hydroelectric plant. The scheduling spans 24 intervals, considering the valve-point loading effect on thermal generators. The input data, including bus data, PDF parameters, and cost coefficients, were gathered from a preceding study^30^. Notably, during intervals 11, 12, and 13, peak loads are identified, prompting DSM to redistribute 10% of the load from these hours to the 2nd, 3rd, and 4th intervals. It's important to note that the pumped-storage hydroelectric (PSH) plant operates in generating mode specifically when both the power generated and discharge rate are positive. Conversely, it functions in pumping mode when pumping power and pumping rate are negative^30^.

**Figure 4:**
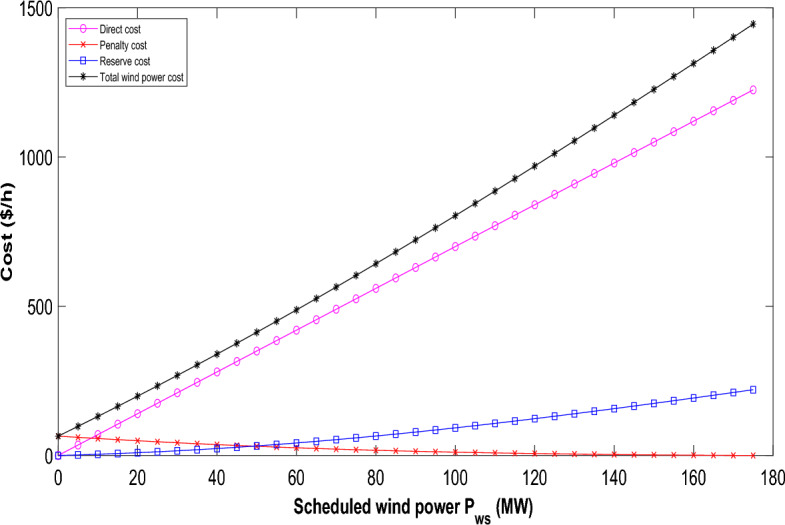
Variation in the cost of wind power relative to scheduled power for wind generators.

**Figure 5:**
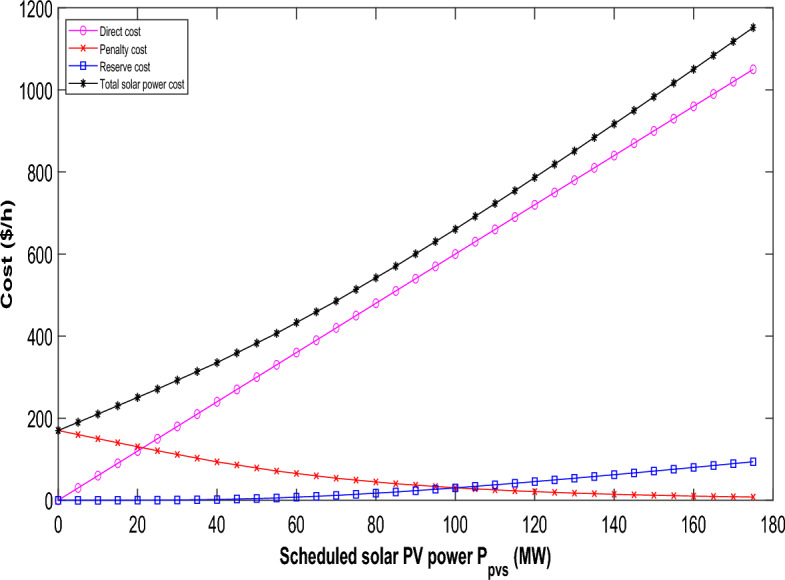
Fluctuation in the cost of solar power versus scheduled power for solar PV units.

The Weibull PDF parameters in this case are chosen from Ref.^30^. The direct cost coefficients, penalty cost coefficients, and reserve cost coefficients for wind power are sourced from literature^30^. Notably, the direct cost of renewable power is lower than the average cost of thermal power. Additionally, the penalty incurred for underutilizing available wind power is less than the direct cost^40^. Examining the scheduled power range from 0 to the wind farm's rated power, Fig. 4 illustrate the variations in reserve, penalty, direct, and total costs for the two wind farms. The total cost comprises the combined direct, reserve, and penalty costs corresponding to the scheduled power. The direct cost shows a linear relationship with scheduled power; as scheduled power rises, a larger spinning reserve becomes necessary, leading to increased reserve costs and consequently driving up the overall generation cost. The penalty cost decreases, albeit at a slower rate, as scheduled power increases. Similarly, the cost variations for solar power over/under-estimation against scheduled power are portrayed in Fig. 5. The yearly operating and maintenance costs for solar PV power plants align within a comparable range to those of onshore wind power plants^30^. Lognormal PDF parameters for solar irradiance are adopted from Ref.^30^ as well. Furthermore, the direct cost coefficients, penalty cost coefficient, and reserve cost coefficient for solar power are also referenced from literature^30^. Yet, using the chosen PDF parameters for solar irradiance, the overall cost of solar power doesn't follow a strictly upward trajectory.

**Figure 6:**
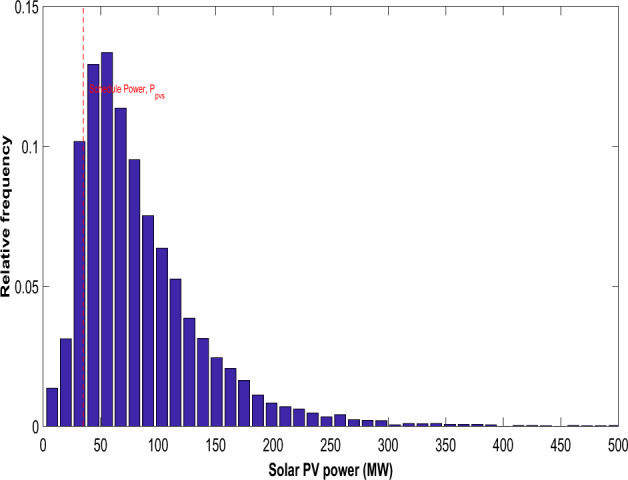
Distribution of real power (MW) from solar PV.

**Figure 7:**
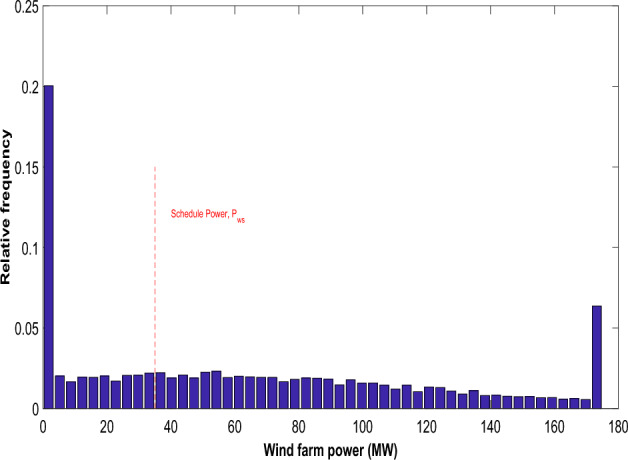
Distribution of real power (MW) from wind farm.

The solar PV plant's stochastic power output is shown as a histogram in Fig. 6. The solar PV system's scheduled electricity delivery to the grid is shown by the red dotted line. As previously said, the schedule power can be any amount of electricity that ISO and the owner of the solar PV firm mutually agreed upon. Figure 7 represents the stochastic power generated by the wind farm. The red dotted line represents the scheduled electricity delivery to the grid by the wind farm. Tables 1 and 2 presents the optimal scheduling of the ten-unit system with and without DSM respectively. The best, average and worst cost and average CPU time among 100 runs of solutions acquired from the proposed ECOA, COA and GWO with and without DSM are summarized in Table 3. It is observed from Table 3 that execution time for ECOA algorithm is lesser compared to COA, GWO, HSPSO, CCDE, FCEP and CFCEP. Reduced computation time enables more effective implementation of demand-side management strategies since quick response times are essential for implementing demand response programs, load shedding, or load shifting, contributing to improved demand-side management and grid reliability. Furthermore, faster computation facilitates better integration of variable renewable energy sources by adapting quickly to their inherent variability.

**Table 1:** Optimal Scheduling of the 10-Unit System to Minimize Operational Costs without DSM.

Hour	$P_{G1}$	$P_{G2}$	$P_{G3}$	$P_{G4}$	$P_{G5}$	$P_{G6}$	$P_{G7}$	$P_{G8}$	$P_{G9}$	$P_{G10}$	$P_{W}$	$P_{PV}$	$P_{GH}$
1	56.369	101.93	76.486	80.001	52.265	68.002	112.16	210.77	202.29	133.42	146.26	0	− 100
2	46.265	113.78	74.847	106.8	94.454	120.01	137.14 5	199.91	189.57	149.41	174.97	0	− 100
3	52.414	112.68	69.1	121.12	81.051	68.034	132.65	145.02	178.07	170.41	169.45	0	− 100
4	36	99.134	60.854	111.65	97	98.343	185.82	138.13	169.51	138.55	175	0	− 100
5	66.575	69.426	78.941	122.58	70.484	92.821	180.76	149.93	161.96	199.06	175	2.4674	− 100
6	72.954	108.92	71.755	122.46	97	122.31	114.96	226.58	135	143.08	175	29.976	− 100
7	78.747	96.444	98.868	80	69.357	127.86	175.26	203.36	150.24	183.31	175	71.568	− 100
8	100.25	114	62.013	94.745	84.438	104.74	115.75	277.86	176.85	132.78	175	101.56	− 100
9	91.245	111.08	60.868	90.157	97	68.185	135.64	286.83	135.1	158.94	77.135	107.82	100
10	108.38	75.323	78.146	122.86	78.229	68	139.67	230.91	156.82	197.33	103.98	130.35	100
11	90.355	109.59	68.657	83.845	95.575	89.456	210.47	279.09	135.07	177.36	40.853	159.69	100
12	107.43	82.915	68.746	84.613	97	104.34	250.11	282.86	177.89	157.24	43.476	153.88	89.4996
13	107.46	89.675	82.648	92.879	69.059	137.39	177.46	215.91	135.8	133.63	126.7	135.28	96.1123
14	96.877	100.77	95.341	127.21	87.245	108.1	144.08	213.47	136.1	160.66	116.57	97.166	16.4035
15	88.568	88.228	94.776	112.25	89.325	91.954	120.18	198.84	137.13	163.38	81.161	87.363	86.8651
16	101.67	103.69	93.559	81.962	91.737	90.376	133.66	142.27	191.47	149.24	175.04	34.157	31.1899
17	114.57	87.975	77.885	125.06	97	119.18	187.05	156.86	150.02	167.37	25.976	40.137	30.9262
18	113.46	95.974	89.964	80.075	83.874	125.16	138.37	181.38	205.68	192.27	67.895	21.178	64.7108
19	114	81.986	76.897	132.86	97	100.13	141.26	157.62	235.66	163.45	154.16	9.2724	75.7171
20	87.264	93.894	60.285	104.74	87.787	139.96	206.25	159.79	284.16	174.53	89.179	0	22.1501
21	84.528	102.89	85.279	114.47	84.436	94.975	256.23	135.02	279.57	157.84	174.76	0	− 100
22	103.67	94.127	93.716	80.093	84.489	82.126	291.74	195.43	222.18	141.72	110.7	0	− 100
23	87.353	112.85	68.025	123.46	75.234	71.983	276.77	135	235.43	138.79	105.1	0	− 100
24	85.143	94.78	60.967	80.835	53.968	84.865	233.28	135.43	263.83	202.79	84.111	0	− 100

**Table 2:** Optimal Scheduling of the 10-Unit System to Minimize Operational Costs with DSM.

Hour	$P_{G1}$	$P_{G2}$	$P_{G3}$	$P_{G4}$	$P_{G5}$	$P_{G6}$	$P_{G7}$	$P_{G8}$	$P_{G9}$	$P_{G10}$	$P_{W}$	$P_{PV}$	$P_{GH}$
1	113.7	83.48	106.1	80	47	135.5	149.9	135	135	145.1	109.2	0	− 100
2	60.68	69.39	120	80.53	72.38	89.99	151	196.5	149	155.5	125	0	− 100
3	114	99.98	74.57	87.96	97	125.1	110.5	202.5	136.5	148.9	103	0	− 100
4	40.02	53.86	71.01	103.9	95.61	130	240.6	131.1	209.2	131	103.8	0	− 100
5	101.31	96.177	60	80	97	93.483	201.58	198.64	135	130.28	175	1.531	− 100
6	102.81	114	61.806	95.321	72.751	124.92	160.41	148.68	191.15	148.88	175	24.27	− 100
7	114	91.751	66.466	108.5	76.599	128.21	123.23	150.68	211.03	190.24	175	74.31	− 100
8	110.92	114	60	80	97	96.356	165.78	139	240.71	151.57	175	109.67	− 100
9	114	88.635	86.921	74.294	96.905	140	110	135.89	137.96	245.44	147.83	116.05	26.077
10	113.85	114	101.02	80	82.989	104.15	184.55	148.97	290.22	135.33	63.874	131.94	39.1097
11	114	82.131	120	81.127	53.703	93.107	170.86	194.29	300	133.02	85.488	164.08	48.2002
12	101.42	114	106.43	80	72.706	68	125.77	145	299.75	272.43	22.025	185.61	106.8507
13	114	78.888	104.91	97.221	94.729	68.875	110	166.9	299.02	275.91	13.601	123.9	52.0403
14	105	44.822	79.712	80	89.245	96.126	180.52	156.42	227.41	135.82	115.46	105.86	83.6024
15	67.591	77.363	89.312	87.174	97	68	110	174.8	151.74	274.83	55.032	87.155	100
16	47.398	88.502	106.37	80	71.112	104.7	126.89	159.42	205.75	186.45	175	34.373	34.0362
17	40.223	114	69.957	118.79	65.547	68	110	197.81	245.52	134.55	171.04	38.106	6.461
18	73.747	86.484	60	158.79	47	80.794	120.09	240.26	205.37	156.99	116.79	21.503	92.1738
19	87.287	114	97.597	131.57	58.785	104.36	110	225.78	267.93	190.99	42.81	8.8921	100
20	114	85.367	74.886	190	75.73	68	149.87	209.86	210.02	182.92	49.344	0	100
21	105.67	114	60	136.68	97	110.69	190.37	231.68	288.45	176.74	58.713	0	− 100
22	77.876	106.99	76.672	141.57	96.762	68	255.35	201.76	236.08	131.4	107.55	0	− 100
23	93.923	82.877	78.265	88.772	91.786	82.301	175.74	195	300	142.37	98.957	0	− 100
24	86.877	70.656	60	80	77.523	80.593	152.24	249.7	238.26	171.97	112.19	0	− 100

**Table 3:** Statistical analysis of optimization results for test system – I.

Algorithm	Minimum operating cost ($)	Mean operating cost ($)	Maximum operating cost ($)	Standard deviation cost ($)	Execution time (s)
With DSM					
CFCEP^30^	3,87,732	3,87,735	3,87,741	NA	23.9351
FCEP^30^	3,88,213	3,88,218	3,88,226	NA	31.5054
CCDE^30^	3,88,309	3,88,314	3,88,324	NA	33.1036
HSPSO^30^	3,88,322	3,88,330	3,88,342	NA	37.0679
GWO^53^	3,87,635	3,87,639	3,87,642	NA	24.6821
COA	3,87,609	3,87,614	3,87,625	0.374	23.8047
ECOA (Proposed)	3,87,595	3,87,603	3,87,615	0.185	21.1208
Without DSM					
CFCEP^30^	3,88,651	3,88,655	3,88,662	NA	22.3517
FCEP^30^	3,89,059	3,89,064	3,89,073	NA	30.0548
CCDE^30^	3,89,158	3,89,165	3,89,174	NA	32.5302
HSPSO^30^	3,89,207	3,89,215	3,89,225	NA	35.9527
GWO^53^	3,88,566	3,88,571	3,88,578	NA	23.6924
COA	3,88,539	3,88,545	3,88,555	0.384	22.5736
ECOA (Proposed)	3,88,525	3,88,533	3,88,545	0.128	20.3891

**Figure 8:**
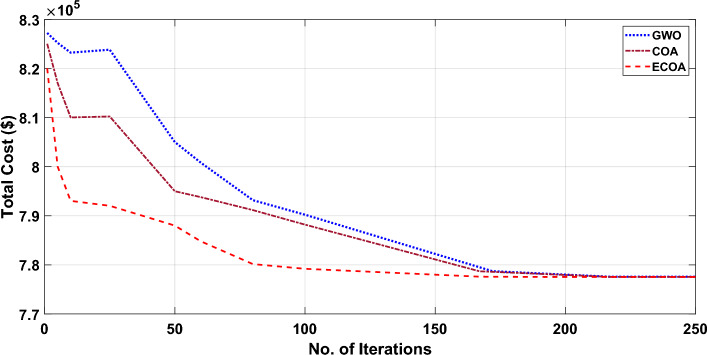
Characteristics of convergence in a 10-unit system without DSM.

**Figure 9:**
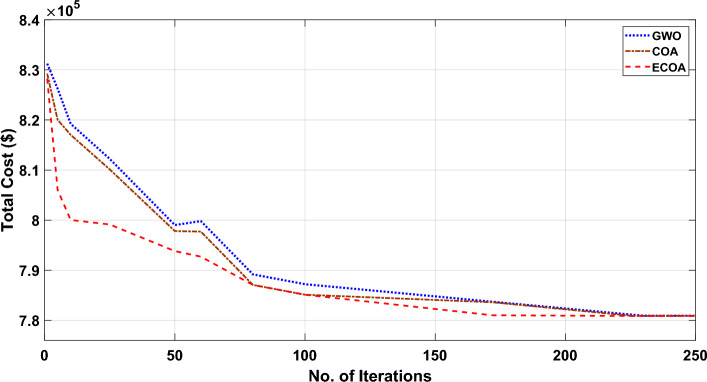
Characteristics of convergence in a 10-unit system with DSM.

Figures 8 and 9 illustrate the cost convergence patterns obtained from the proposed ECOA, COA, and GWO algorithms, both with and without DSM.

It is apparent from Fig. 8’s convergence characteristics that the proposed ECOA algorithm achieves convergence after 163 iterations in the context of dynamic economic dispatch with demand-side management. In comparison, the conventional COA and GWO algorithms converge at the end of 167 and 172 iterations, respectively. It is evident from Fig. 8 that the convergence behavior indicates the proposed ECOA algorithm reaches convergence after 170 iterations, while the conventional COA and GWO algorithms converge at the conclusion of 173 and 180 iterations, respectively. The findings suggest that the convergence of the proposed ECOA was not only swift but also exhibited a smoother trajectory compared to COA and GWO. Table 3 reveals that the operational cost is minimized when dynamic economic dispatch incorporates Demand-Side Management (DSM), as opposed to dynamic economic dispatch without DSM. Furthermore, the cost derived from the proposed ECOA remained the most economical among all methods. The achieved mean cost value closely approached the minimum, showcasing ECOA's competence in reaching global optimal solutions. Moreover, Table 3 reveals that the proposed ECOA algorithm exhibits a lower standard deviation in comparison to COA and GWO. This reduced standard deviation suggests greater stability and consistency in the performance of the ECOA algorithm, highlighting its potential for reliable and predictable outcomes. Due to space limitations, results acquired from COA and GWO^53^ cannot be given here. A sensitivity analysis was performed based on 100 trial test runs. Table 4 displays the results of the sensitivity analysis conducted for the proposed ECOA algorithm applied to Test System I and II. The results lead to the conclusion that a population size of 30 for the provided test system yields the global optimum for the test system—I. Consequently, the simulation outcomes firmly support the conclusion that the ECOA algorithm, as introduced in this study, holds significant potential for delivering high-quality solutions when contrasted with alternative algorithms.

**Table 4:** Sensitivity Analysis for the test systems I and II.

Algorithm	Population size					
	10	20	30	40	50	60
Test System—I (With DSM**)**						
COA	4,46,643	4,46,834	**3,87,609**	3,95,344	3,98,753	4,14,628
ECOA (Proposed)	4,41,012	4,42,982	**3,87,595**	3,94,746	3,97,534	4,10,122
Test System—I (Without DSM)						
COA	4,32,143	4,12,484	**3,88,539**	3,94,213	3,99,322	4,16,454
ECOA (Proposed)	4,29,832	4,10,242	**3,88,525**	3,92,332	3,94,354	4,07,323
Test System—II (With DSM)						
COA	8,64,213	8,45,288	8,14,386	**7,77,552**	7,86,344	8,85,645
ECOA (Proposed)	8,60,435	8,39,334	8,04,537	**7,77,537**	7,84,242	8,78,256
Test System—II (Without DSM)						
COA	8,75,534	8,41,898	8,17,747	**7,80,897**	7,95,586	8,89,528
ECOA (Proposed)	8,73,638	8,39,575	8,10,686	**7,80,884**	7,92,672	8,76,821

Significant values are in [bold].

### Test system: II

This system comprises twenty thermal power plants, two similar wind power generation units, two equivalent solar photovoltaic (PV) facilities, and two pumped-storage hydroelectric plants. The data for this test system are derived by mirroring the information from test system 1. Notably, the power demand in this configuration is twice that of test system 1. Specifically, hours 11, 12, and 13 represent peak load periods. During Demand-Side Management (DSM), 10% of the load during the 11th, 12th, and 13th hours is shifted to the 2nd, 3rd, and 4th hours. The optimal scheduling of the 20-unit system with and without DSM respectively for the 20-unit system is presented in Tables 5 and 6. Tables 5 and 6 provides an analysis of the best, average, and worst costs and average CPU time for 100 runs of solutions obtained from the proposed ECOA, COA and GWO with and without DSM. Table 7 reveals that the computational time for the ECOA algorithm is notably shorter than that of COA, GWO, HSPSO, CCDE, FCEP, and CFCEP algorithms. This accelerated computational speed enables swift decision-making in the face of dynamic system conditions, including abrupt shifts in demand or renewable energy generation. Additionally, the proposed ECOA algorithm adeptly harnesses available renewable energy while safeguarding system stability, thereby optimizing the equilibrium between conventional and renewable generation. Furthermore, faster algorithms may require fewer computational resources, making them more efficient and cost-effective for implementation on various hardware platforms, including embedded systems or edge devices.

**Table 5:** Optimal scheduling of the 20-unit system to minimize operational costs without DSM.

$Hour$	$P_{G1}$	$P_{G2}$	$P_{G3}$	$P_{G4}$	$P_{G5}$	$P_{G6}$	$P_{G7}$	$P_{G8}$	$P_{G9}$	$P_{G10}$	$P_{G11}$	$P_{G12}$	$P_{G13}$
1	108.1029	60.1923	60	80	65.7863	97.6835	110	243.5302	145.2416	152.3126	83.3058	114	60
2	91.0981	36	63.7358	120.6764	47	99.5283	136.876	268.6854	135	130	102.5503	88.656	63.2285
3	73.1295	62.1047	66.5134	104.0168	48.5109	68	152.8058	231.3161	138.095	147.1419	114	114	60
4	67.9991	61.5119	64.7481	143.5745	47	85.821	140.2162	173.3276	135	130	112.9842	104.4644	97.4367
5	55.6974	56.509	60	177.062	71.6839	68	110	141.3841	193.2149	181.3715	114	92.4425	107.5309
6	44.6613	36	96.4745	134.3154	47	101.5172	130.6187	183.95	224.7853	135.5623	109.524	92.9528	86.1058
7	36	47.8664	120	190	53.1942	68	110	135	187.9087	182.5007	114	77.3611	120
8	73.6868	36	80.7531	171.8098	47	83.0275	146.9289	206.5279	135	156.5039	94.1844	114	87.4071
9	36	37.9839	108.6906	149.5987	49.6962	69.2374	110	193.3063	202.5991	130	114	97.7036	120
10	100.123	75.1051	120	108.1616	54.7552	68	165.2875	264.2586	259.2461	135.0203	103.8804	114	81.0339
11	75.7331	84.9877	92.6434	143.843	59.2309	114.2713	173.4729	300	252.1301	130	114	95.2477	60
12	84.4967	114	60.6099	154.0953	47	68	220.019	249.6406	278.8747	186.6605	91.0165	114	90.4077
13	69.1382	78.4242	60	113.3671	72.1971	92.2096	195.6303	215.8091	251.3734	141.3433	68.9178	81.2684	92.2367
14	88.9082	39.8821	64.4997	111.7048	75.0528	68	184.3167	249.2074	174.928	170.7527	92.6253	114	60
15	110.8295	42.1354	60	80	47	98.8403	175.3106	300	135	138.4318	78.7269	110.7036	72.041
16	91.2153	84.9405	66.1724	102.4882	75.9566	68	115.5887	281.1194	150.5427	154.0954	39.487	108.3686	60
17	38.9726	38.0418	88.0523	84.1843	97	79.5784	110	228.1104	221.6327	132.5589	39.3133	78.9023	64.63
18	36	46.8847	60	127.9403	92.7418	68	189.4286	287.822	265.7663	160.604	62.6874	114	97.5153
19	63.4276	78.7592	70.2449	80	97	86.1347	110	244.202	263.8824	170.2554	82.2628	80.5542	64.8019
20	36	114	60	102.1573	87.574	88.2215	112.1325	164.829	212.2056	231.9443	114	41.632	64.1221
21	75.0578	109.423	88.6467	80	97	68	110	238.3878	221.6501	176.3759	107.3378	36	77.9447
22	36	105.323	120	103.0307	84.0713	107.3123	128.7362	213.149	188.2462	130	114	39.6238	69.2848
23	37.0253	88.2791	113.8247	80	70.2716	93.9824	196.5583	290.224	173.6683	141.358	97.3037	65.9074	73.0128
24	36	114	105.5582	121.169	52.0558	75.6393	163.4923	215.3294	138.8152	130	74.4361	76.183	60

$Hour$	$P_{G14}$	$P_{G15}$	$P_{G16}$	$P_{G17}$	$P_{G18}$	$P_{G19}$	$P_{G20}$	$P_{W1}$	$P_{w2}$	$P_{PV1}$	$P_{PV2}$	$P_{GH1}$	$P_{GH2}$
1	80	47	68	110	177.1258	135	145.2984	168.7103	168.7103	0	0	− 100	− 100
2	84.3366	58.1412	70.1596	132.8084	135	179.2514	196.1092	150.5794	150.5794	0	0	− 100	− 100
3	81.6938	79.3056	68	110	183.0599	157.7592	190.5473	175	175	0	0	− 100	− 100
4	80	93.6586	83.87	167.1174	179.8135	135	206.2304	155.1132	155.1132	0	0	− 100	− 100
5	95.1894	97	68	181.5469	179.5619	141.6913	195.8725	175	175	1.1209	1.1209	− 100	− 100
6	124.2447	74.0307	95.2349	110	211.5975	172.0199	206.1434	175	175	36.6309	36.6309	− 100	− 100
7	113.2291	97	68	121.2502	284.3411	229.0946	183.0757	175	175	66.0891	66.0891	− 100	− 100
8	155.373	95.5338	110.8291	110.6361	217.1306	242.9829	148.2919	175	175	108.1967	108.1967	− 100	− 100
9	132.3259	97	138.1668	115.0341	176.8372	219.3531	150.0817	102.4631	102.4431	114.679	114.671	65.2941	92.8352
10	80	96.9051	128.9554	169.9397	209.8324	270.244	163.2031	70.0916	70.0966	135.0605	135.0605	1.0251	0.7143
11	105.4322	70.2628	81.9824	110	262.9495	256.2991	131.5255	72.8291	72.8091	161.8973	161.8973	65.8765	30.6793
12	131.7218	97	68	184.4151	218.9225	296.1273	194.0114	24.414	24.415	156.209	156.209	69.4251	20.3088
13	80	82.7181	97.8308	110	169.4343	300	163.1071	126.2277	126.2277	117.2148	117.2093	83.9202	94.1948
14	83.2266	97	75.0409	149.3401	135.9134	254.3718	195.7902	92.5371	92.5371	103.9221	103.9221	22.5207	100
15	135.6014	74.0102	68	110	148.6189	176.5508	182.4218	75.7884	75.7884	94.9903	94.9903	94.2207	100
16	81.3946	97	81.0251	115.7603	176.2134	135	165.3819	175	175	35.763	35.763	68.7235	100
17	90.2805	74.0399	80.7472	148.6343	135	160.5651	166.4329	175	175	33.4296	33.4296	86.4635	100
18	127.402	97	69.3392	200.3352	202.0722	210.5119	159.3673	80.3367	80.3367	23.8616	23.8616	35.1226	1.0621
19	80	86.8416	110.2013	230.3272	225.14	170.1286	225.3737	165.1055	165.7055	0.3878	0.3878	87.377	41.4986
20	118.1765	82.2929	140	281.0238	257.9843	229.77	236.5814	73.625	73.625	0	0	98.1028	100
21	125.2106	97	137.7453	232.1439	300	270.7243	201.5218	144.9152	144.9152	0	0	− 100	− 100
22	97.4715	94.3273	140	300	234.9859	288.2082	201.8611	102.1844	102.1844	0	0	− 100	− 100
23	141.6286	93.037	113.6687	228.0614	237.2991	300	169.1697	27.8599	27.8599	0	0	− 100	− 100
24	122.1083	97	86.2949	193.8073	264.365	270.0783	237.082	63.2929	63.2929	0	0	− 100	− 100

**Table 6:** Optimal scheduling of the 20-unit system to minimize operational cost with DSM.

$Hour$	$P_{G1}$	$P_{G2}$	$P_{G3}$	$P_{G4}$	$P_{G5}$	$P_{G6}$	$P_{G7}$	$P_{G8}$	$P_{G9}$	$P_{G10}$	$P_{G11}$	$P_{G12}$	$P_{G13}$
1	37.3128	113.8076	97.9928	80.0952	96.9905	68	175.9137	146.9246	167.0984	130.9087	36.9984	36.0176	93.5046
2	36	74.5683	119.907	89.2318	95.0956	101.2094	152.9806	154.5984	141.7792	184.5178	63.4197	62.9975	119.984
3	36.1197	112.8643	110.3084	81.7605	96.9014	131.5096	161.7571	135.8964	135.0853	131.0968	62.4208	95.148	106.5183
4	38.4291	88.5094	74.6193	90.4389	70.0581	139.7603	112.0816	198.0846	136.3087	205.4107	102.1917	101.4295	119.8981
5	36.0378	64.2761	60.0678	81.8924	47.0873	122.6198	158.5708	135.9815	135	157.1624	114	74.2763	110.5423
6	60.5084	102.2803	68.4642	120	54.4903	139.9728	110.0284	186.4127	197.6057	130	84.1791	81.9235	104.3547
7	36.0061	113.9284	60.1784	79.9823	47.0289	140	162.4973	135	239.9571	161.1096	114	114	95.654
8	39.4973	114	70.1287	115.6082	65.1803	139.8702	110	194.1197	216.3743	130	75.9201	89.606	120
9	36.1823	113.9835	88.9051	79.9851	47.1204	94.3806	117.371	135	239.0358	135.684	109.0012	67.7355	61.1097
10	67.0164	82.0184	60.2149	110.1268	54.7403	101.4972	110	206.0016	253.5906	143.5232	114	44.6623	120
11	89.5683	72.1096	87.6046	79.9653	70.6208	76.8705	136.6458	135	300	130	78.5271	36	100.5328
12	113.9805	93.4121	119.9706	102.9037	96.9825	115.0291	110	190.3009	235.7152	136.3359	114	63.1466	74.4287
13	86.8703	113.9786	120	80.0631	81.9317	68.0289	174.7342	135	209.5424	130	92.0238	36	103.3732
14	103.9038	84.5189	115.8753	126.9403	67.8047	100.4103	164.309	174.4941	217.245	199.975	114	72.0433	101.4909
15	101.2608	113.8728	117.3586	110.0237	82.7104	68.0375	110	242.7881	295.8536	130	75.2704	36	82.4004
16	97.4271	103.2914	110.0648	93.8236	87.6302	68	116.5027	192.3857	235.5748	148.084	88.6398	36.8851	84.6239
17	73.8902	113.9734	87.9256	80.0317	84.3516	68.1034	110	135	218.3567	130	97.3548	36	60
18	106.0299	80.1178	71.0974	97.2408	92.5073	107.6023	122.3339	187.9961	240.8119	178.3037	92.8131	66.4119	86.3314
19	113.1892	113.9841	80.0873	0.49503	96.9861	73.4058	140.4095	202.2621	243.3256	130	83.5021	97.722	60
20	53.2831	85.0235	64.9074	101.7126	95.1689	93.5189	209.1839	156.9075	300	166.5225	114	114	69.786
21	113.589	113.9748	86.7583	80.0638	96.9827	139.8923	226.012	135	277.9724	197.6992	104.9794	107.8184	60
22	83.9741	110.0328	71.6073	86.8309	97	134.1082	250.296	164.683	300	130	114	114	74.2884
23	111.3018	113.7943	69.5485	80.1074	96.9572	135.5708	195.3541	135	236.4314	131.077	91.3774	79.6101	60
24	82.8706	89.7031	67.2091	123.6673	85.4982	139.9984	212.4477	196.901	229.1149	130	99.1642	114	80.4807

$Hour$	$P_{G14}$	$P_{G15}$	$P_{G16}$	$P_{G17}$	$P_{G18}$	$P_{G19}$	$P_{G20}$	$P_{W1}$	$P_{w2}$	$P_{PV1}$	$P_{PV2}$	$P_{GH1}$	$P_{GH2}$
1	80.9865	65.3868	135.7805	162.5987	299.9175	222.4703	145.7904	42.807	42.698	0	0	− 100	− 100
2	134.9542	54.1173	139.0863	124.6997	260.1784	250.0169	220.9632	143.9145	143.7784	0	0	− 100	− 100
3	188.5076	47.9876	102.1566	149.1296	297.9851	253.9012	152.9458	175	175	0	0	− 100	− 100
4	133.1037	71.8045	85.0932	199.5053	267.8705	180.7985	174.6051	175	175	0	0	− 100	− 100
5	80	97	117.8084	158.6468	238.736	239.1221	144.1009	175	175	8.5371	8.5371	− 100	− 100
6	85.5447	80.554	72.987	160.7096	188.0746	233.6558	155.6407	175	175	36.3077	36.3077	− 100	− 100
7	138.0218	97	68	216.4846	187.7225	202.1911	130.7652	175	175	65.2387	65.2387	− 100	− 100
8	100.6637	82.0863	78.9181	261.0531	255.0128	152.8245	148.1213	161.2237	161.1237	99.3363	99.3363	− 100	− 100
9	80	71.9432	68	254.7606	220.8481	149.0445	224.0972	146.7796	147.2496	114.5869	114.5869	81.395	41.2139
10	85.3087	47	97.1716	298.3814	188.7563	135	181.8477	108.8248	108.7248	136.4118	136.4118	88.7667	100
11	84.9246	64.217	68	245.5182	135	135.8734	228.0353	39.8849	39.8049	165.5604	165.5604	86.1753	100
12	88.3885	90.1177	123.071	198.5515	173.113	135.012	200.7906	32.4445	32.0545	154.3801	154.3801	47.9464	63.5473
13	80	74.9642	68	110	149.726	135	157.0464	119.355	119.355	138.6669	138.6669	68.4906	89.1846
14	114.0378	47	96.0641	165.5976	142.7678	191.4358	144.6205	103.6662	103.6662	97.0957	97.0957	51.2425	2.7032
15	80	69.4132	140	110	174.7635	135	182.6264	71.8047	71.9247	96.3721	96.3021	70.2559	15.9605
16	126.8792	58.5364	111.9376	130.9252	135	142.1664	145.0151	175	175	37.143	37.143	65.4135	36.9085
17	127.8299	97	113.4723	110	144.0982	135	130.4953	175	175	43.2377	43.2377	70.6439	100
18	84.1304	80.1879	130.585	178.8923	136.2552	173.9859	230.7839	128.7198	128.7198	24.8131	24.8131	30.428	38.0889
19	106.2855	87.4505	140	154.6307	295.6345	178.6413	193.8302	160.821	160.921	2.0812	2.0812	66.6929	95.5607
20	160.6522	73.2388	95.4266	180.8051	245.2812	169.7054	165.757	77.807	77.807	0	0	49.5079	100
21	146.4634	71.9394	80.0493	161.2413	205.8004	240.8837	142.8759	175	175	0	0	− 100	− 100
22	184.9398	47	115.8709	188.1886	214.9894	163.6858	140.3121	107.0969	107.0969	0	0	− 100	− 100
23	171.5641	72.9698	137.6944	191.1575	204.1894	239.0902	140.4945	83.3546	83.3546	0	0	− 100	− 100
24	113.1805	47	100.8866	139.4604	135	300	203.1523	35.1323	35.1323	0	0	− 100	− 100

**Table 7:** Statistical analysis of optimization results for test system—II.

Algorithm	Minimum operating cost ($)	Mean operating cost ($)	Maximum operating cost ($)	Standard deviation cost ($)	Execution time (s)
With DSM					
CFCEP^30^	7,77,681	7,77,686	7,77,693	NA	35.0743
FCEP^30^	7,78,212	7,78,217	7,78,227	NA	44.9034
CCDE^30^	7,78,323	7,78,333	7,78,344	NA	47.0977
HSPSO^30^	7,78,371	7,78,383	7,78,393	NA	48.5723
GWO^53^	7,77,583	7,77,589	7,77,596	NA	34.8702
COA	7,77,552	7,77,557	7,77,568	0.354	33.9125
ECOA (Proposed)	7,77,537	7,77,542	7,77,553	0.105	32.7846
Without DSM					
CFCEP^30^	7,80,948	7,80,954	7,80,962	NA	33.3047
FCEP^30^	7,81,265	7,81,271	7,81,279	NA	43.0541
CCDE^30^	7,81,337	7,81,346	7,81,358	NA	45.1057
HSPSO^30^	7,81,399	7,81,410	7,81,423	NA	45.9378
GWO^53^	7,80,921	7,80,927	7,80,939	NA	33.3047
COA	7,80,897	7,80,903	7,80,914	0.398	32.4092
ECOA (Proposed)	7,80,884	7,80,890	7,80,902	0.092	31.2318

**Figure 10:**
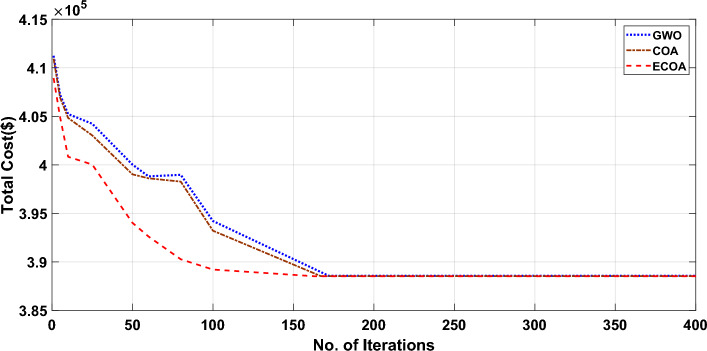
Characteristics of convergence in a 20-unit system without DSM.

**Figure 11:**
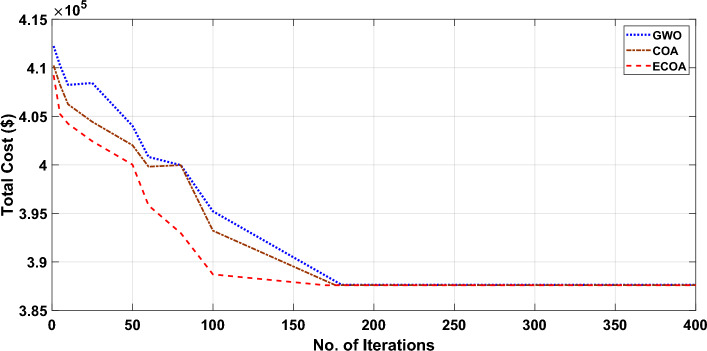
Characteristics of convergence in a 20-unit system with DSM.

Figures 10 and 11 show the cost convergence characteristics obtained from planned ECOA, COA, and GWO^54^ with and without DSM respectively. It is evident from Fig. 10’s convergence characteristics that the proposed ECOA algorithm achieves convergence after 221 iterations, while the conventional COA and GWO algorithms converge at the end of 226 and 230 iterations, respectively. It is noted from Fig. 11 that the convergence characteristics indicate the proposed ECOA algorithm achieves convergence after 193 iterations in the scenario of dynamic economic dispatch with demand-side management. In contrast, the conventional COA and GWO algorithms converge at the conclusion of 215 and 217 iterations, respectively. According to the findings, the proposed ECOA's convergence characteristic was faster and smoother than those of COA and GWO. Table 6 showcases that the inclusion of DSM results in lower costs compared to scenarios without DSM. Furthermore, among all the approaches, the proposed ECOA exhibits the most economical cost. The achieved mean cost value was close to the lowest value. Table 7 illustrates that, in comparison to COA and GWO, the proposed ECOA algorithm displays a diminished standard deviation. This decrease in standard deviation implies enhanced stability and consistency in the performance of the ECOA algorithm, underscoring its potential for delivering reliable and predictable outcomes. This proves that ECOA has the efficacy to create a global optimal solution. The findings from COA and GWO cannot be presented here due to space restrictions. The outcomes of the sensitivity analysis for the proposed ECOA algorithm on Test System I and II are presented in Table 4. These results indicate that, for Test System II, a population size of 45 results in the global optimum. Based on the simulation outcomes, it is evident that the ECOA algorithm proposed in this study possesses a greater probability of generating superior-quality results compared to alternative algorithms.

## Conclusion and future research directions

The current study introduces an enhanced Cheetah Optimizer Algorithm that addresses the unpredictability of renewable energy sources and the involvement of pumped-storage hydroelectric units. This enhancement serves as a practical solution for real-life Distributed Energy Dispatching (DED) scenarios, both with and without Demand-Side Management (DSM). The proposed ECOA, COA and GWO are used to resolve two test systems. Optimization results indicate that the operational expenses associated with Demand-Side Management (DSM) are lower compared to those incurred without its implementation. Furthermore, research indicates that the introduced ECOA algorithm surpasses COA and GWO in performance metrics. The proposed ECOA approach exhibits adaptability and reliability, making it a viable solution for tackling multi-objective energy management challenges within a microgrid, especially when integrating demand response mechanisms. Future endeavors will involve exploring the capabilities of the enhanced cheetah optimization algorithm in addressing multi-objective optimization problems that encompass constraints. This investigation will specifically focus on navigating the trade-offs between conflicting objectives and constraints. Additionally, there is an opportunity to delve into hybridization with other optimization techniques, aiming to enhance convergence speed and improve solution accuracy. The suggested ECOA can be analyzed for its application in realizing the multi-objective optimal operation of bipolar DC microgrids. Furthermore, the suggested ECOA can be applied to elucidate the multi-objective dynamic optimal power flow problem in multi-microgrid systems which involve the integration of electric vehicles and renewable energy sources.

## Data Availability

The datasets used and/or analysed during the current study available from the corresponding author on reasonable request.

## List of symbols

$F\left( {P_{G} } \right)$: Total generating cost
$$t$$: Time
$P_{Wi}$: Power generated by the *i*th wind power generating unit
$P_{PVi}$: Power generated by the *i*th solar power generating unit
$P_{GHi}$: Power generated by the *i*th pumped hydroelectric storage unit
$P_{loss}$: Active power loss
$C_{W}$: Direct cost function for wind farm
$C_{PV}$: Direct cost function for solar photovoltaic generation
$C_{{R_{W} }}$: Reserve cost for the wind unit
$C_{{R_{PV} }}$: Reserve cost for the solar photovoltaic generation
$P_{W r,i}$: Rated wind power of *i*th wind power generating unit
$P_{PV r,i}$: Rated wind power of *i*th solar power generating unit
$F_{C}$: Overall operation cost
$P_{Gi}$: Real power output of *i*th generator
$$P_{D}$$: Power demand
$P_{loss}$: Active power loss
$N_{TH}$: No. of thermal power generating units
$N_{W}$: No. of wind power generating units
$N_{PV}$: No. of solar power generating units
$N_{pump}$: No. of pumped hydroelectric storage units
$T_{pump}$: Collection of time intervals during which the pumped-storage plant operated in pumping mode
NB: Total number of buses
$DR_{t}$: Percentage of the predicted base load involved in Demand Response Participation (DRP) at time t.
$Inc_{t}$: Quantity of added load at time t
$Ls_{t}$: Load that can be shifted at time
$L_{Base, t}$: Predicted base load at time t
$v_{in}$: Cut-in wind speed
$v_{out}$: Cut-out wind speed
$v_{r}$: Rated wind speed
$a_{i} ,b_{i} \;{\text{and}}\;c_{i}$: Fuel cost coefficients of *i*th generating unit
$e_{i} \;{\text{and}}\;f_{i}$: Fuel cost coefficients of *i*th generating unit with valve point effect
$Q_{gj}$: Reactive power output at jth bus
$B_{jk}$: Transfer susceptance between bus j and bus k
$G_{jk}$: Transfer conductance between bus j and bus k
h: Hour
DED: Dynamic economic dispatch
DSM: Demand side management
ELD: Economic load dispatch
OPF: Optimal power flow
COA: Cheetah optimizer algorithm
ECOA: Enhanced Cheetah optimizer algorithm
GWO: Grey wolf optimizer
PSH: Pumped-storage hydropower
ENSCSO: Enhanced non-dominated sorting crisscross optimization
ADFA: Ameliorated Dragonfly algorithm
CFCEP: Chaotic fast convergence evolutionary programming
FCEP: Fast convergence evolutionary programming
CCDE: Colonial competitive differential evolution
HSPSO: Heterogeneous strategy particle swarm optimization
SGEO: Social group entropy optimization
TG: Thermal generator
PDF: Probability density function
POZ: Prohibited operating zone
PV: Photovoltaic
DG: Distributed generation
WT: Wind turbine
NA: Not available
UR: Upward ramp
DR: Downward ramp
DR: Demand response
TOU: Time-of-use
PSO: Particle swarm optimization
ISO: Independent system operator
